# Genetic variation and evolutionary relationships of dominant ticks in Northwestern Iran utilizing COI and ITS2

**DOI:** 10.1007/s00436-026-08650-0

**Published:** 2026-02-18

**Authors:** Meysam Motevasseli, Eslam Moradi-Asl, Parisa Soltan-Alinejad

**Affiliations:** Arthropod-Borne Diseases Research Center, Ardabil University of Medical Sciences, Ardabil, Iran

**Keywords:** Hard ticks, Mitochondrial DNA, COI, ITS2, Genetic diversity, Phylogenetic analysis

## Abstract

Hard ticks are important vectors of medical and veterinary pathogens. Accurate species identification is crucial for understanding the dynamics of tick-borne diseases, yet the presence of cryptic species, damaged specimens, or immature stages often limits morphological methods. This study investigated the genetic diversity and phylogenetic relationships of ticks in northwestern Iran. In total 283 ticks collected from domestic livestock across ten towns in Ardabil Province. Morphological identification was complemented by molecular analyses using mitochondrial COI and nuclear ITS2 markers. Phylogenetic trees were constructed using Maximum Likelihood. Genetic distances were estimated with the Kimura two-parameter (K2P) model. Morphological and molecular analyses identified three species: *R. sanguineus* (*n* = 132), *H. marginatum* (*n* = 80), and *D. marginatus* (*n* = 71). COI and ITS2 markers reliably discriminated species and revealed frequent misidentification between *R. sanguineus* and *R. turanicus*. ITS2 haplotypes of *R. sanguineus* matched populations from Italy and Israel, whereas COI data showed high genetic similarity among local and Asian populations. Both markers showed that *H. marginatum* and *D. marginatus* clustered with Turkish lineages. Molecular markers and haplotype analyses provide robust tools for accurate species identification, revealing tick diversity, population structure, and dispersal pathways. These insights are essential for effective surveillance and control of tick-borne diseases in the region.

**Supplementary Information:**

The online version contains supplementary material available at 10.1007/s00436-026-08650-0.

## Introduction

Hard ticks, after mosquitoes, serve as important carriers of medical and veterinary pathogens worldwide (Ali et al. 2020). Ticks serve as vectors for numerous pathogens and parasites that are important to the health of humans, domestic animals, and wildlife (Al-Deeb et al. 2015). Identifying tick species accurately is crucial for the control of ticks and tick-borne diseases (Apanaskevich and Horak 2008; Claerebout et al. 2013; Dantas-Torres et al. 2013). Traditionally, this relies on the morphology of adults; however, it becomes problematic with damaged, blood-engorged, or subadult specimens (larvae and nymphs) (Caporale et al. 1995; Guglielmone et al. 2006). Furthermore, relying solely on morphological methods can create difficulties in identifying cryptic species or specimens with similar features (Szabó et al. 2005; Guglielmone et al. 2014; Nava et al. 2014). Molecular techniques that analyze DNA sequence variation provide a more precise and objective way to assess genetic diversity within and between species, thereby overcoming previous limitations (Cruickshank 2002). Numerous factors, including seasonal migration, the presence of hosts, the ecological demands of various developmental stages, and the dispersal abilities of ticks, are thought to impact the divergence, genetic adaptation, and variability of ticks, which may eventually contribute to genetic differentiation and speciation (Ali and Mulenga et al. 2020). To evaluate the genetic variability both within and between parasitic populations, it is crucial to comprehend their dispersal and transmission dynamics (Kanduma et al. 2016).

Different types of genetic markers, encompassing both coding and non-coding areas, can be utilized in phylogenetic studies. Mitochondrial coding genes, including COI, 12 S rRNA, and 16 S rRNA, are commonly utilized in phylogenetic studies of ticks, providing enhanced resolution at both the genus and species levels (Black 4th and Piesman 1994; Murrell et al. 2000; Beati and Keirans 2001). The cytochrome c oxidase I (COI) mitochondrial gene is a universal molecular identifier for DNA barcoding (Hebert et al. 2004; Hajibabaei et al. 2006). Non-coding markers commonly used in phylogenetic analyses include the internal transcribed spacers ITS1 and ITS2 of the nuclear ribosomal DNA (rDNA). Among these, the ITS2 region has been widely applied in phylogenetic studies of diverse tick species (Hlinka et al. 2002). The investigation focused on the genetic variation and phylogenetic ties of hard ticks in northwestern Iran by sequencing and analyzing mitochondrial COI and nuclear ribosomal ITS2 markers, as well as comparing the sequences obtained with those from other regions.

## Methods and materials

### Study area and sample collection

The samples were collected from ten towns across Ardabil province from June to September 2024: Ardabil, Parsabad, Aslanduz, Bilehsavar, Germi, Khalkhal, Nir, Namin, Saryin, and Kowsar. Hard ticks were obtained from domestic livestock such as sheep, cattle, and goats found in animal shelters. Specimens were delicately removed with forceps from the abdomen, neck, throat, and limbs to ensure their integrity was preserved. The ticks were stored in 75% ethanol for subsequent analysis. Tick species were identified based on a standard morphological taxonomic key (Hosseini-Chegeni et al. 2019).

### DNA extraction, PCR amplification, and DNA sequencing

Tissue samples were individually homogenized in liquid nitrogen. Genomic DNA was subsequently extracted from each sample using the FavorPrep^®^ Tissue DNA Extraction Kit (FAVORGEN, Iran), following the manufacturer’s protocol. The ITS2 region was amplified from the extracted DNA in a PCR reaction using Taq DNA Polymerase Master Mix RED (Ampliqon^®^, Denmark) and the specific primers DITS2-F (5′-GTGCGTCCGTCGACTCGTTTTGA-3′) and DITS2-R (5′-ACGGCGGACTACGACGGAATGC-3′) (Hlinka and Murrell et al. 2002; Soltan-Alinejad et al. 2020). The PCR thermal profile comprised an initial denaturation at 95 °C for 5 min, followed by 30 amplification cycles of 95 °C for 30 s, 68 °C for 30 s, and 72 °C for 30 s, with a final extension at 72 °C for 5 min. Similarly, a fragment of the COI gene was amplified using the universal primers LCO (5′-GGTCAACAAATCATAAAGAGATTGG-3′) and HCO (5′-TAAACTTCAGGGTGCCCAAAAAATCA-3′) (Folmer et al. 1994). The PCR conditions for COI were: initial denaturation at 94 °C for 5 min; 30 cycles of 94 °C for 30 s, 48.5 °C for 30 s, and 72 °C for 30 s; with a final extension at 72 °C for 7 min. The expected amplicon sizes were 710 bp for COI and 610 bp for ITS2. The nucleotide sequences of the PCR amplicons were determined by Sanger sequencing.

### Sequence analyses and phylogenetic tree

The sequences of COI and ITS2 were compared with the data present on GenBank via the Blast tool on the NCBI website (https://blast.ncbi.nlm.nih.gov/Blast.cgi) after the low-quality sequences were trimmed from both ends. Highly similar reference sequences were obtained from GenBank and utilized for genetic analyses, the construction of phylogenetic trees, and the computation of genetic distances. For each tick species, a minimum of two specimens were chosen based on the molecular analysis of their COI and ITS2 sequences. Phylogenetic relationships of ticks were inferred using the Maximum Likelihood method based on the Tamura-Nei model (Tamura and Nei 1993). Initial trees for the heuristic search were obtained automatically by applying Neighbor-Join and BioNJ algorithms to a matrix of pairwise distances estimated with the Maximum Composite Likelihood (MCL) approach, with the topology exhibiting the highest log likelihood value being selected. A discrete Gamma distribution was used to model evolutionary rate differences among sites. All analyses were conducted in MEGA12 software (Kumar et al. 2024). The robustness of the phylogenetic trees was assessed using a bootstrap test with 1,000 replicates, with values shown at the branch points. The sequence of *Argas persicus* was included as an outgroup for phylogenetic tree reconstruction.

### Genetic distance

Kimura two-parameter (K2P) pairwise distances were computed for COI and ITS2 sequences. Genetic distances among individuals were estimated using the Kimura two-parameter (K2P) model (Kimura 1980) (Kimura 1980), as implemented in MEGA 12 to assess sequence divergence.

## Result

### Morphological and molecular characterization of tick species using nucleotide BLAST

**Fig. 1:**
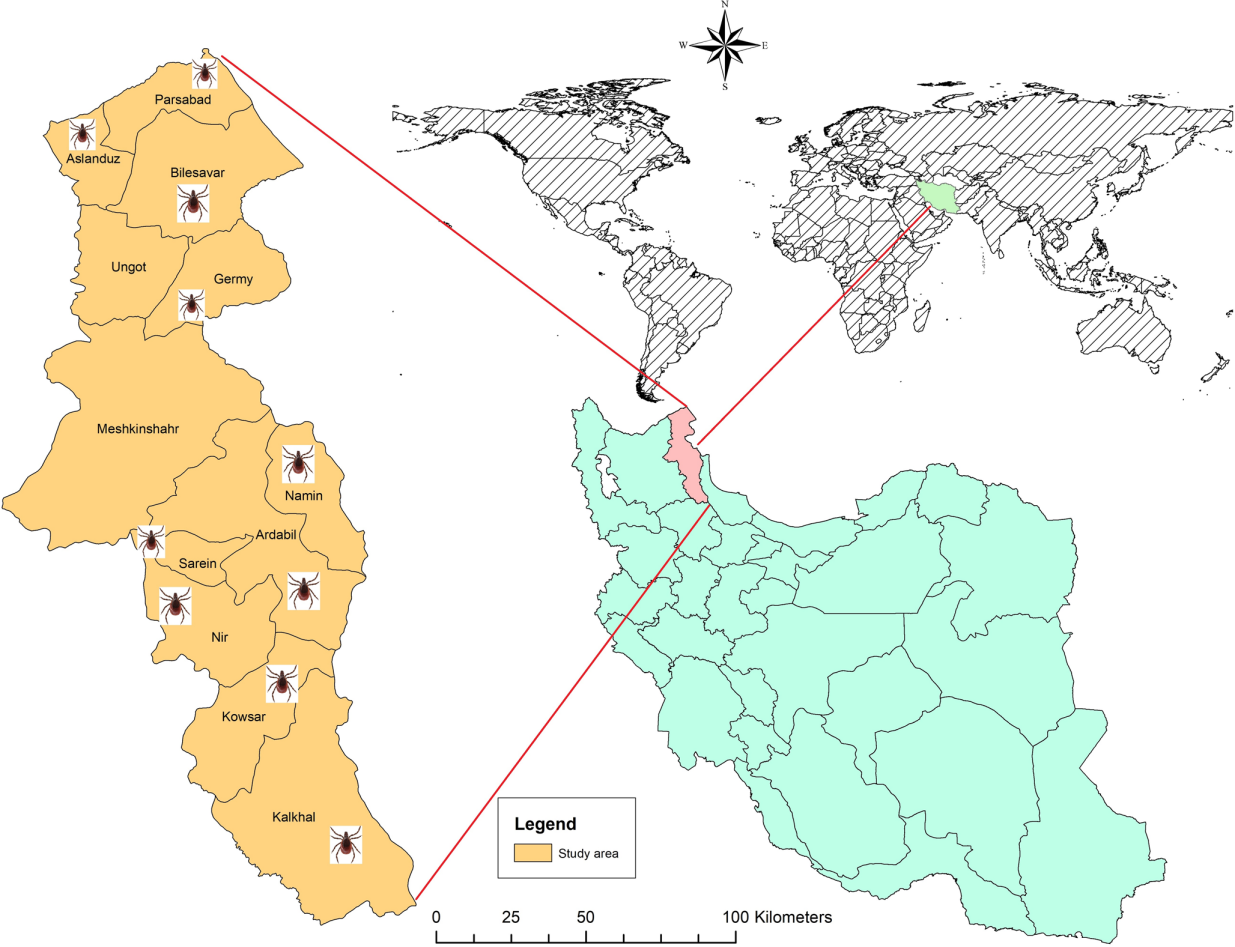
Map showing the geographical location of the surveyed sites from which ticks were collected in the study area

As shown in Table 1, a total of 283 ticks were collected from the examined animals. The geographical location of the surveyed sites is shown in Fig. 1. Of these, 71 specimens were identified as *Dermacentor marginatus*, 80 as *Hyalomma marginatum*, and 132 as *Rhipicephalus sanguineus* based on standard identification keys. Following DNA extraction and initial PCR, 12 samples that demonstrated optimal quality and quantity for both genetic markers (COI and ITS2) were selected for sequencing. Consequently, sequence data for both gene regions were obtained from the same set of samples. This selection was made to minimize errors and enhance the reliability of the subsequent phylogenetic analyses.

**Table 1:** Distribution and number of ticks collected from examined animals at different surveyed sites

County	Location	*D. marginatus*	*H. marginatum*	*R*. *sanguineus*	Total (%)
Ardabil	**48.29425767° E** **38.24700867° N**	**17**	**4**	**21**	**52 (18.02)**
Aslanduz	**47.410188° E** **39.441331° N**	**21**	**0**	**2**	**22 (7.77)**
Bilehsavar	**48.34771367° E** **39.38249567° N**	**0**	**6**	**19**	**25 (8.83)**
Parsabad	**48.28031833° E** **37.79835733° N**	**8**	**1**	**14**	**15 (5.3)**
Germi	**48.076227° E** **39.041364° N**	**0**	**6**	**14**	**20 (7.06)**
Khalkhal	**48.52463267° E** **37.62137567° N**	**15**	**0**	**20**	**35 (12.36)**
Nir	**48.01312667° E** **38.03606767° N**	**0**	**22**	**15**	**38 (13.28)**
Namin	**48.482288° E** **38.42627° N**	**2**	**30**	**0**	**32 (11.3)**
Saryin	**48.07284867° E** **38.14553067° N**	**0**	**1**	**17**	**18 (6.36)**
Kowsar	**48.27511167° E** **38.19728667° N**	**8**	**10**	**10**	**28 (9.89)**
Total (%)	**-**	**71 (25.08)**	**80 (28.26)**	**132 (46.64)**	**283**

Morphological identification *of D. marginatus*,* H. marginatum*, and *R. sanguineus* was confirmed by GenBank BLAST analysis of both COI and ITS2 sequences, revealing 98–100% sequence identity with the corresponding species. The sequences generated in this study have been deposited in GenBank under accession numbers PX695456–PX695467 for the COI gene and PX696054–PX696063 for the ITS2 gene.

The COI sequences of *R. sanguineus* from this study showed the highest similarity (97–99.25%) to sequences of isolates from other parts of Iran and Kazakhstan (GenBank: OR533795.1). Based on the COI gene BLAST analysis, these specimens showed a close relationship to *R. turanicus* reported from Albania (GenBank: KY606298.1), with a sequence similarity of 97%. The COI sequences of *H. marginatum* obtained in this study showed 100% similarity to isolates reported from Turkey (GenBank: PV019968.1 and MW366632.1) and France (GenBank: PV019815.1). Furthermore, COI sequences of *D. marginatus* obtained from Ardabil were identical (100% similarity) to reference sequences from Turkey (GenBank: OP581266.1, OP581277.1, and PP455526.1) and 99% sequence similarity with Kazakhstan (GenBank: MN964340.1).

Regarding the ITS2 sequences, analysis of *R. sanguineus* from the present study revealed the highest similarity (98–99%) to *R. turanicus* isolates from Italy (GenBank: KF499552.1) and Israel (GenBank: KF958426.1), as well as to *R. sanguineus* reported from Iran (GenBank: KT313077.1). The COI sequences of *H. marginatum* generated in this study showed 98–99% similarity to isolates reported from Turkey (GenBank: MW508348.1) and Iran (GenBank: KP208953.1, KP208954.1, and KP208964.1). In addition, ITS2 sequences of *D. marginatus* from Ardabil exhibited 98–99% similarity to reference sequences from Turkey (GenBank: PP456850.1 and PP456846.1) and Iran (GenBank: KJ00439.1).

### Phylogenetic analysis and genetic distances using the COI marker

**Fig. 2:**
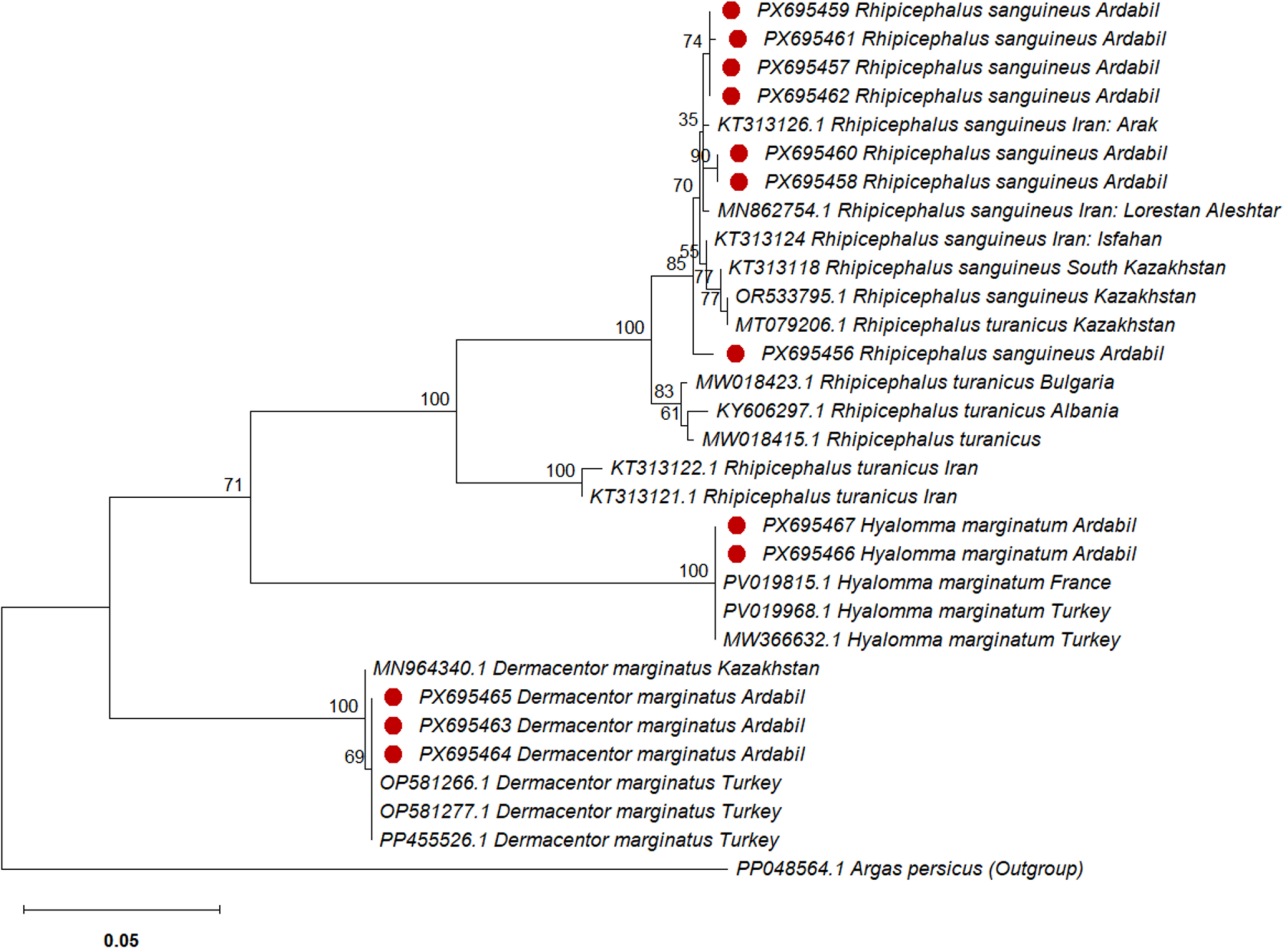
Phylogenetic relationships for DNA sequences of 710 bp of cytochrome Oxidase I (COI) region among 31 populations of 4 species belonging to 3 genera of hard ticks. (Red circle indicates the sequences obtained in the current study)

Figure 2 shows the phylogenetic relationships among the studied tick populations based on 710 bp COI sequences. Distinct clades were identified in the COI-based phylogenetic tree corresponding to *R. sanguineus*,* R. turanicus*,* D. marginatus*,* and H. marginatum*, confirming that COI effectively discriminates tick species at both the genus and species levels. The mean K2P distances at the intraspecific level ranged from 0 to 0.01, whereas the interspecific distances between *R. sanguineus* and *R. turanicus* ranged from 0.02 to 0.10. All *R. sanguineus* ticks collected in the present study clustered into a single clade together with *R. sanguineus* isolates from other provinces of Iran and Kazakhstan. In this tree, the sequence MT079206.1, recorded as *R. turanicus* from Kazakhstan, clustered within the *R. sanguineus* clade. In the COI gene analysis, the results provided strong support (100%) for a cluster comprising *R. sanguineus* specimens from Iran, as well as those of Asian origin, including Kazakhstan. Additionally, a close phylogenetic relationship was observed between *R. sanguineus* and *R. turanicus* specimens from European regions, specifically Bulgaria and Albania. To evaluate the discriminatory power of the COI marker at the species level, genetic distances were calculated between the two Kazakhstan sequences, MT079206.1 and OR533795.1. The results showed zero genetic divergence (K2P = 0) between these sequences. This finding indicates that the sequence MT079206.1 was morphologically misidentified and incorrectly deposited in GenBank, further confirming the accuracy and reliability of the COI marker for species-level identification of ticks. The low intraspecific K2P distances (0–0.01) indicate high genetic similarity among individuals of the same species, whereas the markedly higher interspecific distances (0.02–0.10) between *R. sanguineus* and *R. turanicus* demonstrate clear genetic divergence, supporting the effectiveness of the COI marker in discriminating closely related tick species. Notably, the highest genetic divergence for this gene (K2P: 0.09–0.10) was observed at the interspecific level, occurring between European *R. turanicus* and Iranian *R. turanicus* (GenBank: KT313121.1 and KT313122.1) populations. *H. marginatum* specimens identified from Ardabil Province (GenBank: PX695467 and PX695466) clustered within the same clade as *H. marginatum* sequences reported from Turkey (GenBank: PV019968.1 and MW366632.1) and France (GenBank: PV019815.1). Analysis of genetic distances among these samples revealed no detectable genetic divergence (K2P = 0). *D. marginatus* specimens identified from Ardabil Province (GenBank: PX695463–PX695465) clustered within the same clade as *D. marginatus* sequences reported from Turkey (GenBank: OP581266.1, OP581277.1, and PP455526.1). Genetic distance analysis revealed no detectable divergence between the Ardabil and Turkish samples (K2P = 0), whereas a marked genetic divergence was observed between these samples and the sequence MN964340.1 from Kazakhstan (K2P = 0.10). Intraspecific and interspecific genetic distances calculated based on COI for *R. sanguineus*, *H. marginatum*, and *D. marginatus* are available in the supplementary file.

### Phylogenetic analysis and genetic distances using the ITS2 marker

**Fig. 3:**
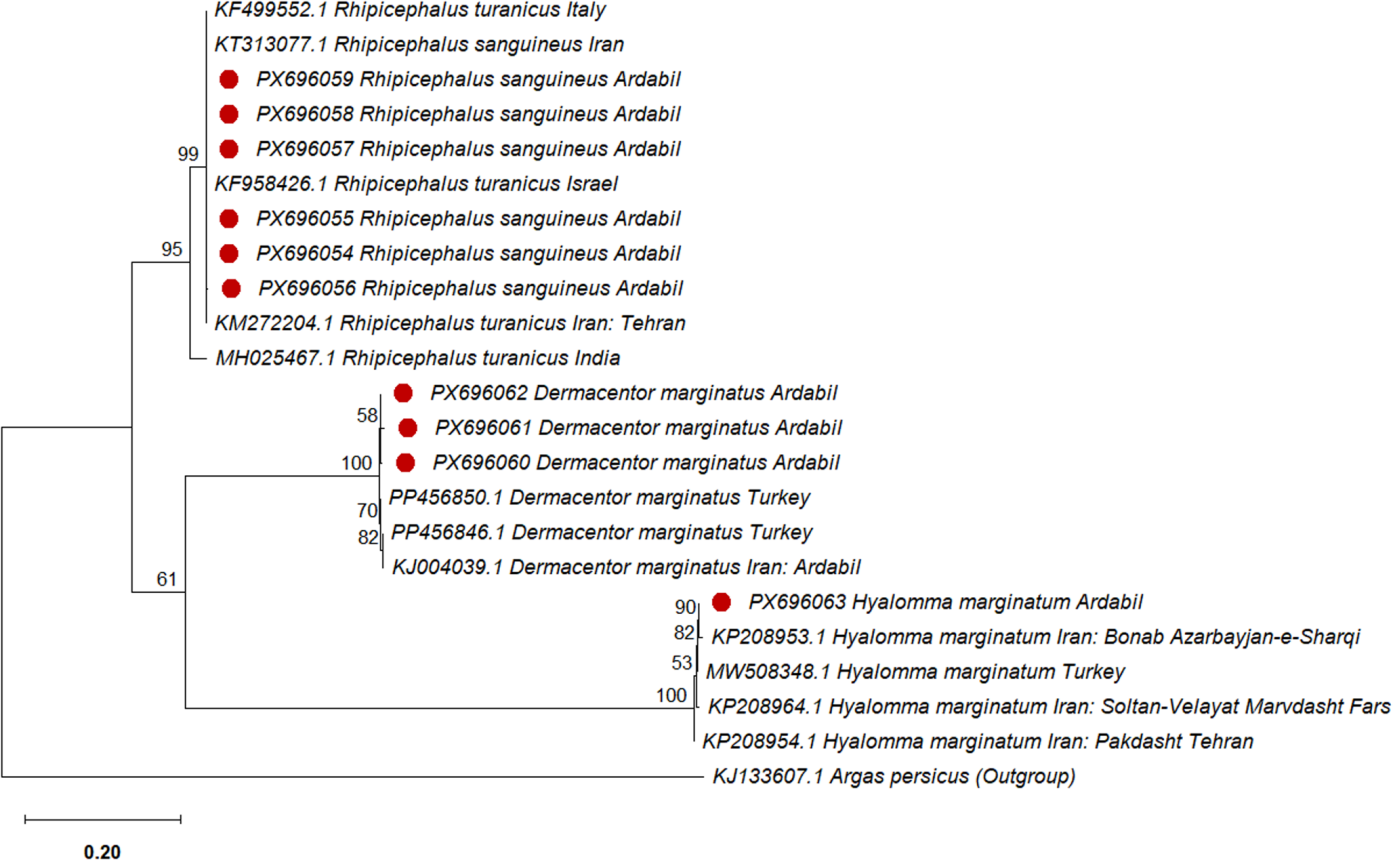
Phylogenetic relationships inferred from DNA sequences of the ITS2 rDNA region (608 bp) among 22 populations representing four species from three genera of hard ticks. (Red circle indicates the sequences obtained in the current study)

Figure 3 shows the phylogenetic relationships among the studied tick populations based on 610 bp ITS2 rDNA sequences. The ITS2-based phylogenetic tree revealed four distinct clades corresponding to *R. sanguineus*,* R. turanicus*,* D. marginatus*, and *H. marginatum*, demonstrating that the ITS2 marker effectively discriminates tick species at both the genus and species levels. The mean K2P distances at the intraspecific and interspecific distances ranged from 0 to 0.04.

In the ITS2-based phylogenetic tree, *R. sanguineus* specimens from Ardabil Province clustered within a single clade along with *R. sanguineus* Iran (GenBank: KT313077.1), *R. turanicus* sequences from Italy (GenBank: KF499552.1), Israel (GenBank: KF958426.1), and Tehran, Iran (GenBank: KM272204.1). In contrast, the sequence *R. turanicus* from India (GenBank: MH025467.1) formed a separate clade. To assess the efficacy of the ITS2 marker for species-level identification in ticks, genetic distances among the sequences were analyzed. The results showed that specimens from Ardabil Province exhibited no genetic divergence (K2P = 0) with *R. sanguineus* from Iran (GenBank: KT313077.1) and *R. turanicus* sequences from Italy (GenBank: KF499552.1), Israel (GenBank: KF958426.1), and Tehran, Iran (GenBank: KM272204.1). One specimen from Ardabil (GenBank: PX696056) exhibited a minor mean genetic difference of 0.003 compared to other samples from this study and specimens from Iran, Italy, and Israel. In contrast, all of the above sequences exhibited a genetic distance of 0.04 from *R. turanicus* from India (MH025467.1). These findings suggest that sequences KF499552.1, KF958426.1, and KM272204.1 may have been morphologically misidentified and incorrectly deposited in GenBank. *D. marginatus* specimens from Ardabil Province clustered within the same clade as conspecific sequences from Ardabil (GenBank: KJ004039.1) and Turkey (GenBank: PP456850.1). Genetic distance analysis based on the K2P model revealed values ranging from 0.007 to 0.01 between specimens from Ardabil and Turkey, whereas lower distances (0.003–0.009) were observed between the specimens collected in this study and other Ardabil specimens. *H. marginatum* specimens from Ardabil Province clustered within the same clade as conspecific sequences from Iran (GenBank: KP208953.1, KP208954.1, KP208964.1) and Turkey (GenBank: MW508348.1). Genetic distance analysis based on the K2P model revealed that the Ardabil specimens showed no genetic divergence (K2P = 0) from KP208953.1 (Iran) and MW508348.1 (Turkey). Intraspecific and interspecific genetic distances calculated based on ITS2 for *R. sanguineus*, *H. marginatum*, and *D. marginatus* are available in the supplementary file.

## Discussion

The *R. sanguineus* group is among the most taxonomically controversial, and species-level discrimination between *R. sanguineus* and *R. turanicus* remains particularly challenging (Al-Deeb and Muzaffar et al. 2015). Clear morphological characters for separating the two species are not available. Consistent with our findings, instances of misidentification between these two species were observed. Importantly, the COI and ITS2 molecular markers demonstrated high discriminatory power, allowing reliable differentiation of ticks at both the genus and species levels. Phylogenetic analyses based on both the COI and ITS2 molecular markers indicate that *R. sanguineus* and *R. turanicus* share a common ancestor with another clade. This relationship is strongly supported by a bootstrap value of 95 to 100, indicating a high level of confidence in the inferred phylogenetic clustering. The previous studies are consistent with our findings, showing that *R. sanguineus* and *R. turanicus* are closely related and likely share a common ancestor (Burlini et al. 2010; Dantas-Torres et al. 2024). In support of this complexity, Santos-Silva et al. (2008) analyzed multiple mitochondrial (COII, 12 S rDNA, and control region) and nuclear (28 S rDNA) markers in *R. turanicus* and *R. sanguineus* from Portugal and reported no detectable genetic differentiation between the two taxa (Santos-Silva et al. 2008). Because genetic and population-level variation among ticks may influence vector competence and susceptibility to control measures, including acaricides, accurate characterization of tick populations is of critical importance. Furthermore, understanding the relationships among geographically distinct tick lineages worldwide can provide insight into their evolutionary origins as well as the factors underlying their dispersal and establishment within a country (Burlini and Teixeira et al. 2010). Based on the ITS2 marker, *R. sanguineus* specimens from Ardabil share identical haplotypes with those from Italy and Israel. However, no corresponding haplotypes were observed in the COI dataset, likely reflecting the absence of COI sequences from the Italian and Israeli populations in GenBank. In contrast to *R. sanguineus*, the *H. marginatum* and *D. marginatus* species sampled from Ardabil Province were genetically distinct from those previously reported in Iran. Phylogenetic analyses based on mitochondrial and nuclear markers revealed that the *H. marginatum* and *D. marginatus* populations from the study area are most closely related to Turkish and French (for *H. marginatum*) and Turkish (for *D. marginatus*) lineages, respectively, suggesting these countries as a probable source and indicating historical or recent dispersal events. Low mitochondrial variation is often seen as evidence of recent population expansion. In such cases, rapid demographic growth restricts the time available for new mutations to accumulate. Similar patterns of low nucleotide diversity have been reported in other tick species, such as *D. variabilis*,* R. microplus*, and *R. appendiculatus* (Krakowetz et al. 2010; Amzati et al. 2018; Díaz-Sánchez et al. 2023). These ticks were probably introduced through migratory birds, livestock movements, and other human-mediated activities, with local environmental conditions facilitating their establishment (Perveen et al. 2021; Hoffman et al. 2023).

## Conclusion

Molecular analyses reveal the close relationship and frequent misidentification between *R. sanguineus* and *R. turanicus*, with COI and ITS2 markers providing reliable species discrimination. Analyses indicate connections with populations from Italy and Israel, while *H. marginatum* and *D. marginatus* show the closest affinity to Turkish lineages, suggesting recent introductions. Low mitochondrial variation supports recent population expansion, likely facilitated by migratory birds, livestock movement, and human activities. These findings underscore the value of molecular markers and haplotype analysis for understanding tick diversity, population dynamics, and pathways of dispersal, informing effective surveillance and control strategies.

## Supplementary Information

Below is the link to the electronic supplementary material.

ESM 1 (CSV 4.84 KB)

ESM 2 (CSV 8.20 KB)

## Data Availability

All data are available in the manuscript. The genome sequence data that support the findings of this study are openly available in GenBank of NCBI at (https://www.ncbi.nlm.nih.gov) under the accession no. PX695456, PX695457, PX695458, PX695459, PX695460, PX695461, PX695462, PX695463, PX695464, PX695465, PX695466, PX695467, PX696054, PX696055, PX696056, PX696057, PX696058, PX696059, PX696060, PX696061, PX696062, PX696063.
